# Autochthonous North American Leprosy: A Second Case in Canada

**DOI:** 10.3390/idr13040083

**Published:** 2021-10-22

**Authors:** Prenilla Naidu, Rahul Sharma, Jamil N. Kanji, Vilma Marks, Arienne King

**Affiliations:** 1 Public Health Laboratory, Alberta Precision Laboratories, University of Alberta Hospital, Edmonton, AB T6G 2J2, Canada; Jamil.Kanji@albertahealthservices.ca; 2 Department of Laboratory Medicine and Pathology, Faculty of Medicine & Dentistry, University of Alberta, Edmonton, AB T6G 2R3, Canada; 3 U.S. Department of Health and Human Services, Health Resources and Services, Health Systems Bureau, National Hansen’s Disease Program Laboratory, Baton Rouge, LA 70809, USA; rahirahul24@gmail.com (R.S.); vmarks@hrsa.gov (V.M.); 4 RetroBioTech LLC, St Louis, MO 63146, USA; 5 Division of Infectious Diseases, Department of Medicine, Cumming School of Medicine, University of Calgary, Calgary, AB T2N 4W4, Canada; 6 Department of Pathology & Laboratory Medicine, Cumming School of Medicine, University of Calgary, Calgary, AB T2N 4W4, Canada; 7 Division of Infectious Diseases, Department of Medicine, Faculty of Medicine & Dentistry, University of Alberta, Edmonton, AB T6G 2R3, Canada; arienne.king@albertahealthservices.ca

**Keywords:** leprosy, Hansen’s disease, leprosy in North America, autochthonous leprosy, *Mycobacterium leprae*, leprosy genotyping

## Abstract

Autochthonous leprosy was reported in the Southern USA in 2011 and has comprised an average of 34% of new cases from 2015 to 2020 in that country. We report a similar case in a patient from Western Canada. A 50-year old male patient presented with a four-year history of a chronic rash. Pathology stains revealed acid-fast bacilli prompting specialist referral. Examination was suspicious for leprosy, which was confirmed on slit skin smears and molecular testing. The patient responded well to treatment. Genotypic testing mapped the organism to the 3I-2 SNP type, which is of European origin and is the type found in implicated armadillo species in North America.

## 1. Introduction

Since the closure of the last leprosarium in 1965 in Canada, leprosy is an uncommon diagnosis, with only five cases reported in the country in 2016 and none in 2017 [1,2]. In Canada, this disease is usually imported (diagnosed among individuals who have immigrated to Canada from leprosy-endemic areas). Bonnar et al. [3], reported a case in a non-immigrant Canadian with a travel history to Florida, United States (USA). Autochthonous leprosy was reported from the Southern USA in 2011 [4], comprising an average of 34% of new cases from 2015 to 2020 [5] diagnosed in people with no history of travel to endemic countries. We report a similar case in a patient from Western Canada highlighting the changing risk factors of present-day leprosy in North America with the increasing role of zoonotic transmission of the disease.

## 2. Case

A 50-year-old male presented with a history of chronic reddish macules throughout his body. The first (herald) lesion, which was asymptomatic, appeared on his back four years prior in 2014. He was referred to a dermatologist two years after the herald lesion when similar lesions developed on his legs. Biopsies of the lesions taken in early 2016 were considered to be consistent with granuloma annulare, although the question of leprosy was raised owing to the presence of nerve involvement with patchy perivascular and interstitial lymphocytic infiltrate. The pathology on repeat biopsies three months later was also consistent with granuloma annulare. That same year, the patient developed numbness and tingling in his left foot with oedema. On referral to neurology, he was diagnosed with mononeuritis multiplex after magnetic-resonance and positron emission tomography excluded multiple sclerosis, transverse myelitis, sarcoidosis, and obvious structural abnormalities.

In January 2018, the patient was referred back to the dermatologist as his skin lesions spread to involve his torso and arms. Biopsies at this time were consistent with granuloma annulare or sarcoidal granulomatous reaction. The pathologic diagnosis at the time was sarcoidosis and his serum angiotensin-converting enzyme level was elevated. He was subsequently referred to a third dermatologist who repeated skin biopsies a month later. These showed sarcoidal granulomatous reaction with lymphocytic infiltration. Auramine-rhodamine stains demonstrated 1+ acid-fast bacilli raising the question of lupus vulgaris (cutaneous tuberculosis). Review of the microscopic description showed focal nerve involvement adding the question of infection due to *Mycobacterium leprae*, prompting referral to an Infectious Diseases physician.

His medical history was significant only for coeliac disease. He was on no medications, was a non-smoker, and did not consume alcohol nor use recreational drugs. He was born and raised on a farm in southeastern Alberta, Canada. As a youth, he avidly participated in rodeos and played hockey. He did not travel abroad until reaching adulthood. His travel history includes resort vacations to Mexico and Dominican Republic, a cruise to Turkey and Greece, remote travel to Texas and a vacation in Virgina, USA. He is a hunter and regularly hunts deer and fowl. He has eaten squirrel meat about 20 years ago while on vacation in Virginia. He has no known exposure to armadillos.

**Figure 1:**
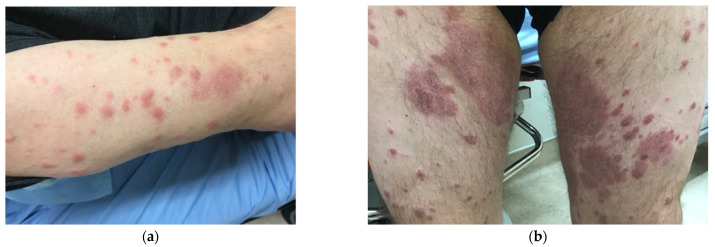

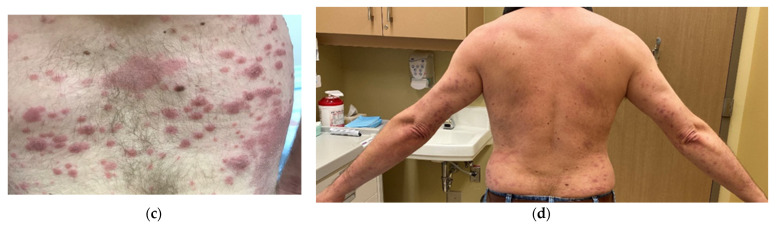
Skin lesions: at diagnosis (**a**) right arm, (**b**) thighs, (**c**) lower back, and post treatment (**d**) arms and back.

On initial examination by Infectious Diseases, he had raised granulomatous lesions on the face, neck, dorsum of his hands, trunk, buttocks, and legs (Figure 1a–c). His palms and the soles were spared. The rash blanched on pressure and areas of confluence had diminished sensation confirmed by pinprick testing. Power and tone were normal in all four extremities. Reflexes were brisk, but symmetrical. The feet were swollen, with diminished pain sensation in a glove and stocking distribution. Diminished sensation to pain and touch were also noted over confluent lesions. The patient reported anhidrosis and numbness in these confluent areas. He had normal proprioception, but decreased vibration sense in the left foot. Multiple skin biopsies from various lesions, nare swabs, and slit skin smears from the earlobes and elbows were obtained.

## 3. Laboratory Results

Serologies for hepatitis C, HIV, Lyme disease, and syphilis were negative. Testing for glucose-6-phosphate dehydrogenase deficiency, Lupus anticoagulant, antiphospholipid antibody and hypercoagulability work up were negative. Complete blood count, urinalysis, liver enzymes, as well as thyroid and renal function tests were within normal limits. C-reactive protein was 13 mg/L (normal <8 mg/L). His tuberculin skin test was negative.

**Figure 2:**
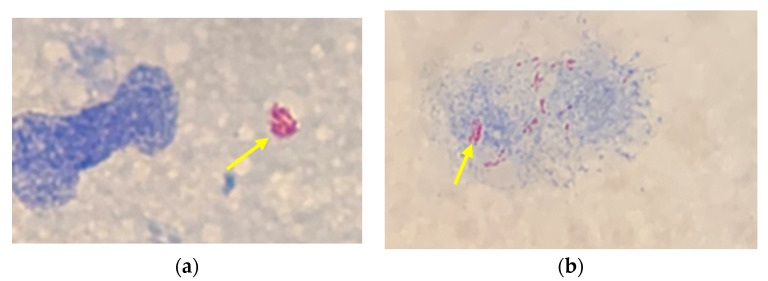
Acid-fast stain of slit skin smears of (**a**) left earlobe and (**b**) left elbow showing AFB-positive bacteria (yellow arrows).

Slit skin smears from both earlobes and elbows had numerous acid-fast bacilli (AFB) (Figure 2a,b), and touch preparations of the skin biopsies of an old lesion from the left upper arm and a new lesion on his neck were both positive for a moderate number of AFB at the local public health laboratory. Nasal scrapings and slit skin smears of the nares were negative for AFB. Direct molecular testing of the tissue specimens was negative for *Mycobacterium tuberculosis* complex on the Xpert MTB/RIF (GeneXpert, Cepheid, Sunnyvale, CA, USA). The tissue biopsies were sent to the National Microbiology Laboratory (Public Health Agency of Canada, Winnipeg, MB, Canada) and to the National Hansen’s Disease Program (NHDP) (Baton Rouge, LA, USA) for confirmation. PCR tests at both laboratories confirmed the presence of *M. leprae*. Based on testing and clinical staging, the patient was diagnosed with lepromatous leprosy.

Single nucleotide polymorphism (SNP) and variable number tandem repeat (VNTR) were performed at NHDP as described [6]. SNP analysis mapped the organism as SNP type 3I-2 of European origin, which is also the only SNP type reported from the armadillos. SNP analysis can link the leprosy strain to the global origin, and, additional analysis of 10 hypervariable VNTR provides strong evidence of the local or zoonotic transmission. Unfortunately, there was insufficient genomic material to complete the VNTR profile to confirm or rule out zoonotic transmission from armadillos in this case.

Two months after start of treatment, repeat slit skin smears of both earlobes and elbows had very few AFB present. Interestingly, right nasal scrapings also contained very few AFB. Ten months after commencement of therapy, repeat slit skin smears, and nasal scrapings were all negative for AFB.

## 4. Case Management and Progress

The patient was started on therapy with rifampin 600 mg monthly, dapsone 100 mg daily, clofazimine 50 mg daily and 300 mg once a month, to be continued until skin smears were negative for two years as per Alberta Health guideines [5]. Public Health advised rifampin prophylaxis for the patient’s household contacts (wife and son) with periodic examinations for skin lesions for 5 years from last contact with the patient [7,8].

The patient tolerated this treatment regimen very well with no Type 1 or Type 2 reactions or significant adverse effects. Besides a mild normocytic anaemia, his blood indices remained within normal limits. Nine months after commencing therapy, the patient’s skin lesions had faded, his face had returned to its normal appearance, and the oedema of his legs and feet was significantly reduced. Neurology evaluation (December 2018) indicated some healing of his affected nerve. The patient completed his therapy in March 2020 and remains well.

## 5. Discussion

Here, we report an autochthonous case of lepromatous leprosy in a Canadian-born male with only short-vacation-related travel to the USA, areas of the European continent, and resorts in Central America. Leprosy is rarely seen in Canada with an estimated prevalence of 0.6 per 100,000 of the population in the last decade (with almost all cases being imported) [9]. Our case represents the second such case, we are able to find in the medical literature, reported from Canada, with a previous case reported in 2014 [3].

The historic route of transmission of *M. leprae* is through close frequent human-to-human contact over several years since humans were thought to be the only host for the bacterium. In the 1970’s, cases of natural infection of the nine banded armadillo with *M. leprae* in Southern USA and Mexico, were discovered. Subsequently, in the 1980’s, natural infection in sooty mangabey monkeys and chimpanzees in Africa, was discovered [10]. Most recently, in the twentieth century, red squirrels in the United Kingdom were found to harbor this bacterium [11,12]. Although *M. leprae* DNA has been detected in soil and water, it has not been cultured from these sources so it is still not clear whether viable *M. leprae* organisms are present in the environment for this to be a potential source of human infection.

The principal risk factor thought to contribute to endemic leprosy acquisition in North America is exposure and zoonotic transmission from armadillos in the southern part of the country [6,13,14]. Interestingly, in one ecologic cohort study conducted at Hansen’s Lab [6], many patients had no recall of contact with armadillos, as in our patient. Leprosy acquired in the United Sates without obvious risk factors (prolonged travel abroad to endemic areas, or zoonotic exposure to wild infected armadillos in Southern USA) has been previously reported, but is uncommon. Four such cases have been reported from New York state [5,13,14,15] and one case from Georgia [16]. However, it is emerging that a more common risk factor for such autochthonous cases is transmission via droplets or aerosols from foreign-born individuals residing in North America from countries with endemic *M. leprae* infection (described previously in Spain) [5,17]. It is difficult to trace such exposures given the prolonged incubation period of clinical leprosy after an infectious exposure (mean of four years for tuberculoid leprosy and ten years for lepromatous leprosy) [18]. With regard to our patient, his travel history was felt to be of short periods where prolonged exposure is less likely. Local exposure to such imported cases could be possible in Alberta as well and cannot be ruled out, with five cases diagnosed between 1998–2000 (all being imported) [9].

Genotyping of *M. leprae* has enabled current and historic mapping of the transmission and spread of the organism. The strain identified in our patient (SNP type 3I-2) has been found in wild armadillos across Southern USA (Texas, Louisiana, Mississippi, Alabama, Georgia, and Northern Florida), responsible for zoonotic cases of leprosy related to these animals [6,19]. This strain is also prevalent in the North American population (Mexico), and slightly different from the 4P strain pre-dominant in Brazil [6]. One study found that armadillos in Southern USA were more likely to have the 3I-2-v1 subtype, with those in the rest of Florida more likely to harbor the 3I-2-v15 subtype, also found in several human cases [6]. The previous Canadian case reported had a genotype of 3I-2-v1 (with travel to Florida) [3]. Unfortunately, there was insufficient genetic material in the samples to confirm the 3I-2 subtype in our patient. However, based on his travel history, the partial genotyping result from his specimens and data from the literature, it is plausible he may have been exposed to *M. leprae* during his travels in Southern USA and Mexico.

Our patient was treated for lepromatous leprosy based on the protocol outlined by the World Health Organisation (WHO), which advises treatment for up to one year [20]. However, as he continued to display AFB-positive skin slits at eight months post-initiation of therapy, a clinical decision was made to extend the treatment for a total of 24 months (using the duration guidance in the NDHP treatment guidelines) and local provincial public health treatment recommendations [9,21]. This decision was made due to concerns of clinical relapse with the 12-month WHO regimen, which has been described [22]. In retrospect, however, this may not have been required as multiple follow-up studies have demonstrated low rates of relapse with the 12-month regimen which are similar to relapse rates seen with a 24-month regimen [23,24]. The probability of relapse, much like the risk of progression to infection after exposure to *M. leprae*, is likely related to genetic components that are not entirely understood [25,26].

Leprosy was eliminated as a global public health problem in 2000 and in most countries by 2006 [27]. As the prevalence of leprosy decreased, the cost effectiveness of maintaining active surveillance systems decreased, resulting in the WHO replacing active with passive surveillance in 2006 [28]. This system relies on symptomatic patients presenting to healthcare providers to initiate the public health response of treatment, contact tracing and monitoring. The WHO Enhanced global strategy for further reducing the disease burden due to leprosy, 2011–2015, focused on early detection to reduce disabilities due to leprosy and the Global Leprosy Strategy 2016–2020 focuses on vulnerable populations, strengthened referral systems, contact tracing, drug resistance monitoring, and the role of post-exposure prophylaxis [27]. In Canada, leprosy is a notifiable disease relying on passive reporting of suspected and confirmed cases from physicians and clinical laboratories [1,9]. This system depends on clinical suspicion and referral to Infectious Diseases physicians for diagnosis and management. The occasional publication of case reports of leprosy can serve to raise awareness amongst physicians [29,30].

## 6. Conclusions

Leprosy is rare in the Canadian-born population and thus not commonly included in differential diagnoses for relevant presentations. Our case highlights the importance of increasing education to clinicians regarding risk factors and changing epidemiology of *M. leprae* acquisition, leprosy presentation, and importance of including it in differential diagnoses as zoonotic transmission is proving to play an increasing role in North America. While transmission of *M. leprae* from imported cases in non-endemic areas has been identified, the significance of this is not yet clearly known.

## Data Availability

Not applicable.
